# Asparagine Synthetase Deficiency: A Novel *ASNS* Variant and a Systematic Review of Clinical Phenotypes and Cerebrospinal Fluid Asparagine Levels

**DOI:** 10.3390/biom16101377

**Published:** 2026-09-22

**Authors:** Patryk Lipiński, Sandra Górska, Julia Brąszkiewicz, Małgorzata Rydzanicz, Rafał Płoski

**Affiliations:** 1 Third Department of Pediatrics, Center of Postgraduate Medical Education, 05-092 Dziekanów Leśny, Poland; 2 Institute of Clinical Sciences, Maria Skłodowska-Curie Medical Academy, 03-411 Warsaw, Poland; sandra.gorska@uczelniamedyczna.com.pl (S.G.); julia.braszkiewicz45527@uczelniamedyczna.com.pl (J.B.); 3 Department of Medical Genetics, Medical University of Warsaw, 02-106 Warsaw, Poland; malgorzata.rydzanicz@wum.edu.pl (M.R.); rploski@wp.pl (R.P.)

**Keywords:** asparagine synthetase deficiency, *ASNS*, cerebrospinal fluid, asparagine, developmental and epileptic encephalopathy, microcephaly, cerebral atrophy, exome sequencing

## Abstract

Background: Asparagine synthetase deficiency (ASNSD) is a rare autosomal recessive neurometabolic disorder associated with microcephaly, severe developmental impairment, epilepsy, and progressive cerebral atrophy. Reduced cerebrospinal fluid (CSF) asparagine has been proposed as a supportive biochemical marker, but the extent to which it provides useful diagnostic information remains uncertain. Methods: We report a female patient with molecularly confirmed ASNSD and conduct a systematic review of molecularly confirmed cases in accordance with PRISMA 2020. PubMed/MEDLINE, Scopus, and Web of Science Core Collection were searched from inception to 20 September 2026. Clinical, survival, neuroimaging, molecular, and CSF-asparagine data were extracted for individual patients whenever available. Outcome frequencies were calculated only among patients for whom the relevant feature was reported. Results: The patient developed infantile spasms at 5 months of age after an initially unremarkable brain MRI and subsequently showed drug-resistant epilepsy, profound developmental impairment, progressive microcephaly, spastic tetraparesis, feeding difficulties, and severe cortical and subcortical atrophy. Exome sequencing identified compound-heterozygous *ASNS* variants: the novel early frameshift variant c.81_82del (p.Gly28SerfsTer7) and the previously reported c.97C>T (p.Arg33Cys), confirmed *in trans*. The systematic review included 106 published molecularly confirmed patients. Developmental impairment was reported in 101/101 assessable patients, microcephaly in 99/106, epilepsy in 72/86, and at least 48 deaths were documented. Exact individual CSF asparagine concentrations were available for only six patients; five were reduced or undetectable and one was within the local reference interval. Conclusions: The novel c.81_82del variant expands the molecular spectrum of ASNSD. The available evidence does not permit calculation of a validated diagnostic threshold, sensitivity, specificity, or predictive values for CSF asparagine. A reduced concentration may support the diagnosis, whereas a normal concentration does not exclude ASNSD. Molecular testing remains the diagnostic standard and should be considered early in patients with developmental and epileptic encephalopathy, progressive microcephaly, and cerebral atrophy, even when early MRI or routine metabolic investigations are unrevealing.

## 1. Background

Asparagine synthetase deficiency (ASNSD; OMIM #615574) is a rare autosomal recessive neurometabolic disorder caused by biallelic pathogenic variants in *ASNS*. This gene encodes asparagine synthetase (EC 6.3.5.4), which catalyses the ATP-dependent synthesis of asparagine from aspartate and glutamine. ASNSD was first delineated by Ruzzo et al. in 2013 in nine children from four unrelated families who presented with congenital microcephaly, severe developmental impairment, and progressive encephalopathy [1].

Since its original description, the recognized clinical and genetic spectrum of ASNSD has expanded substantially. The most frequently reported manifestations include congenital or early postnatal microcephaly, profound developmental impairment, early-onset epileptic encephalopathy, progressive spasticity, and neuroimaging abnormalities, particularly cerebral atrophy [2,3,4,5,6]. Nevertheless, considerable phenotypic variability has become apparent, particularly with respect to the timing of seizure onset, progression of microcephaly, epilepsy severity, and radiological evolution.

The central nervous system appears particularly vulnerable to impaired asparagine biosynthesis because transport of asparagine across the blood–brain barrier is limited, increasing neuronal dependence on local de novo synthesis [1,7]. Consequently, *ASNS* dysfunction predominantly affects brain development and function, resulting in severe neurological manifestations that may begin prenatally and continue to progress throughout infancy and childhood [1,4,8].

Reduced or undetectable cerebrospinal fluid (CSF) asparagine concentrations were initially proposed as a biochemical hallmark of ASNSD [9]. Subsequent reports, however, documented normal or only borderline-reduced concentrations in molecularly confirmed patients [5,10,11,12,13], raising the question of whether, and to what extent, CSF asparagine measurement can provide useful diagnostic information in ASNSD.

We report a female patient with severe developmental and epileptic encephalopathy caused by compound-heterozygous *ASNS* variants, including the previously unreported early frameshift variant c.81_82del (p.Gly28SerfsTer7). We also conducted a systematic review of molecularly confirmed ASNSD to describe the clinical spectrum and assess the available data on CSF asparagine concentrations.

## 2. Materials and Methods

### 2.1. Clinical Evaluation

A retrospective review of all available medical records from birth through the most recent assessment at 11 years of age was performed. Data were collected for the neonatal period, seizure onset at 5 months, neurological and developmental status at 18 months, and the latest follow-up. Clinical, neuroimaging, biochemical, electrophysiological, nutritional, treatment, and molecular findings were reviewed.

### 2.2. Genetic Analysis

The parents elected to pursue exome sequencing (ES) at their own expense. ES was performed in the proband using DNA extracted from peripheral blood and the SureSelectXT Human All Exon V7 enrichment kit (Agilent Technologies, Cedar Creek, TX, USA), using a laboratory workflow previously applied in developmental and epileptic encephalopathy diagnostics [14]. The enriched library was sequenced on a HiSeq 1500 platform (Illumina, San Diego, CA, USA) using 100-bp paired-end reads. The mean sequencing depth was 84×, with 97% and 93% of target regions covered at ≥10× and ≥20×, respectively. Sequencing reads were aligned to the GRCh38 reference genome and analyzed for single-nucleotide variants and small insertions/deletions. Exome-based copy-number variant assessment was not performed as a validated diagnostic component of this analysis; therefore, exon-level or whole-gene *ASNS* deletions/duplications would require targeted CNV testing if clinically suspected.

Variant interpretation was performed according to the American College of Medical Genetics and Genomics and Association for Molecular Pathology (ACMG/AMP) guidelines [15], with computational support from GeneBe (https://genebe.net/, accessed on 18 September 2026) [16]. The PM3 criterion was calibrated according to ClinGen Sequence Variant Interpretation recommendations for variants confirmed *in trans* [17].

Candidate variants were subjected to familial segregation analysis. Targeted testing of the proband’s healthy parents and brother was performed using amplicon-based deep sequencing. Amplicon libraries were prepared using the Nextera XT kit (Illumina) and sequenced using the paired-end protocol described above.

### 2.3. Systematic Review

The review was conducted and reported in accordance with the PRISMA 2020 statement. The protocol was not prospectively registered. PubMed/MEDLINE, Scopus, and Web of Science Core Collection were searched from inception to 20 September 2026 without language restrictions. Search concepts combined the disease and gene terms “asparagine synthetase deficiency”, “ASNS deficiency”, ASNSD, and ASNS with phenotype terms including microcephaly, epileptic encephalopathy, developmental delay, and developmental impairment. Complete database-specific strategies and the record-level screening log are provided in Supplementary Table S3. Reference lists of eligible articles and relevant reviews were searched for additional reports.

Original reports were eligible when they described at least one individual with molecularly confirmed biallelic *ASNS* variants interpreted as pathogenic or likely pathogenic in the source report or supported by the reported phenotype and segregation data. Reviews without additional data on individual patients, experimental studies without clinical cases, monoallelic cases, and reports lacking molecular confirmation were excluded. Potential duplicate publications were reconciled using family structure, country, genotype, age, sex, and clinical features. Two authors independently screened titles and abstracts and assessed full-text articles for eligibility. One author extracted the data, which were independently verified by another author. Disagreements were resolved through discussion and consensus. Extracted items comprised genotype, ancestry/country of origin, sex, age at onset, follow-up or outcome, microcephaly, developmental impairment, epilepsy, tone abnormality, feeding difficulties, neuroimaging, plasma and CSF amino-acid results, and treatment. Each phenotype frequency was calculated using only patients for whom that item was explicitly reported. For CSF asparagine, evidence was classified as: exact quantitative values reported for individual patients; qualitative descriptions for individual patients; cohort-level statements without individual values; or secondary-source-derived statements. No diagnostic-accuracy meta-analysis was planned because thresholds, reference intervals, and complete two-by-two data were unavailable.

The searches identified 171 database records: 60 in PubMed/MEDLINE, 61 in Scopus, and 50 in Web of Science Core Collection. After removal of 87 duplicate records, 84 unique records underwent title and abstract screening; 38 were excluded. Forty-six full-text reports were assessed and seven were excluded with reasons. Thirty-nine database-indexed reports and four additional reports identified through citation and reference-list searching were included, yielding 43 reports in the qualitative synthesis (Figure 1; Supplementary Table S3).

## 3. Results

### 3.1. Case Report

The female patient was born to a non-consanguineous Polish couple following an uneventful second pregnancy. She was delivered spontaneously at 40 weeks of gestation. Her birth weight was 2800 g, length was 54 cm, and head circumference was 31 cm (<3rd percentile). Apgar scores were 10 at both 1 and 5 min.

At 5 months of age, the patient developed epileptic seizures, initially presenting as infantile spasms. Over time, the seizure semiology evolved to include multiple drug-resistant seizure types, including generalized tonic–clonic and myoclonic seizures, which frequently occurred in clusters of several dozen episodes per day. Electroencephalography (EEG) demonstrated generalized epileptiform activity characterized by polyspikes, sharp waves, spike-and-wave complexes, and polyspike-and-wave discharges. Initial brain magnetic resonance imaging (MRI) was unremarkable. Antiseizure treatment was initiated with vigabatrin, followed by valproic acid.

By 18 months of age, the patient had developed progressive microcephaly, generalized axial hypotonia, and severe global developmental delay. She was unable to sit independently. Follow-up brain MRI demonstrated progressive cortical and subcortical cerebral atrophy. Metabolic investigations—including urinary organic acid analysis by gas chromatography–mass spectrometry, amino acid and acylcarnitine profiling in dried blood spots by tandem mass spectrometry, serum transferrin isoelectric focusing, and quantitative plasma amino-acid analysis—yielded results within the relevant reference ranges. The plasma asparagine concentration was normal.

The patient did not attain basic developmental milestones. She remained completely non-verbal, did not establish meaningful eye contact, and failed to develop social communication skills. Progressive microcephaly and profound intellectual disability became increasingly apparent during childhood. Significant feeding difficulties and oral aversion were present from infancy and ultimately resulted in chronic malnutrition. Epilepsy remained drug-resistant, with absent or only transient responses to multiple antiseizure medications, including carbamazepine, lamotrigine, topiramate, lacosamide, and clobazam.

At 11 years of age, the patient weighed 24 kg (<3rd percentile), and her head circumference was 44 cm (<3rd percentile). Neurological examination revealed spastic tetraparesis and an absence of verbal communication and meaningful visual contact. Brain MRI confirmed severe global cortical and subcortical atrophy. EEG monitoring demonstrated persistent generalized epileptiform discharges. Generalized tonic–clonic seizures continued to occur in clusters every 2–3 days. At that time, she was receiving levetiracetam and oxcarbazepine.

Repeated metabolic investigations did not reveal abnormalities suggestive of an inborn error of metabolism. Because of intractable feeding difficulties, a percutaneous endoscopic gastrostomy was placed, and full enteral nutrition was successfully established.

Exome sequencing identified two compound heterozygous variants in *ASNS*. The first was the novel frameshift variant NM_001673.5:c.81_82del, NP_001664.3:p.(Gly28SerfsTer7). The second was the previously reported missense variant NM_001673.5:c.97C>T, NP_001664.3:p.(Arg33Cys) (rs759224338) [18].

The c.81_82del variant is located in exon 3 of 13 and is predicted to introduce a premature termination codon after only six altered amino acids, p.(Gly28SerfsTer7). This very early frameshift is expected to result in nonsense-mediated transcript decay or, less likely, production of a severely truncated non-functional protein lacking the glutamine amidotransferase and asparagine synthetase catalytic domains. The variant was absent from gnomAD v4.1.0 (accessed on 4 December 2025) and was not listed in ClinVar. It was classified as likely pathogenic according to the ACMG/AMP framework based on PVS1, PM2, and PM3 at Moderate strength, because segregation analysis confirmed occurrence *in trans* with a previously reported disease-associated *ASNS* variant. Functional studies were not performed, and this limitation was retained in the final classification.

The c.97C>T variant is extremely rare in gnomAD v4.1.0, with an allele frequency of 0.000004337 (accessed on 4 December 2025), and ClinVar contained two likely pathogenic and one pathogenic submissions (VCV000434402.10; accessed on 7 November 2025). It was classified as likely pathogenic using ACMG/AMP criteria PM2_Supporting, PM3_Moderate, PP3, and PS4_Supporting, based on its rarity, predicted deleterious effect, confirmed occurrence *in trans*, and previous observations in individuals with a concordant ASNSD phenotype.

Familial segregation analysis demonstrated that c.81_82del was inherited from the patient’s healthy mother and was absent in her father and brother. Conversely, c.97C>T was detected in the healthy father and brother but was absent in the mother. These findings confirmed that the variants occurred *in trans*, consistent with autosomal recessive inheritance (Figure 2).

The clinical presentation and molecular findings established a diagnosis of ASNSD.

Quantitative CSF amino acid analysis was not performed because parental consent for lumbar puncture was not provided.

### 3.2. Systematic-Review Findings

The updated review included 106 molecularly confirmed individuals. The original extraction contained 100 individuals from 63 unrelated families (Supplementary Table S1) [1,2,3,4,5,6,7,8,9,10,11,12,13,18,19,20,21,22,23,24,25,26,27,28,29,30,31,32,33,34,35,36,37,38,39,40,41,42,43]. The May 2026 report describing compound-heterozygous c.1031-6_1041del and c.1049A>G variants had already been captured in the original individual-level dataset and was not counted again [44]. The updated searches identified six additional, non-duplicate patients: four affected siblings from Oman with a homozygous *ASNS* splice-region variant, one infant treated with L-asparagine, and one infant with postmortem neuropathological characterization [45,46,47].

Developmental impairment was reported in 101/101 individuals with assessable information, microcephaly in 99/106, and epilepsy in 72/86. Selected clinical findings are summarized in Table 1. These proportions describe reported findings among assessable patients and should not be interpreted as population-based prevalence estimates. Microcephaly was usually congenital and often progressive, but postnatal-onset microcephaly and patients without reported microcephaly demonstrate a broader spectrum. Seizures ranged from absent during reported follow-up to neonatal-onset, drug-resistant developmental and epileptic encephalopathy.

Hypotonia was reported in 32/80 assessable individuals, hypertonia or progressive spasticity in 23/73, and feeding difficulties in 31/57. The published spectrum can be described pragmatically as: (i) prenatal/neonatal lethal or rapidly progressive disease; (ii) severe infantile developmental and epileptic encephalopathy with congenital or progressive microcephaly; and (iii) a less severe childhood-surviving phenotype with later seizure onset, slower progression, or partial developmental acquisition. These categories overlap and are descriptive rather than validated subgroups.

Brain MRI was abnormal in 71/73 individuals with interpretable neuroimaging data. Reported abnormalities included cerebral atrophy, delayed myelination, ventriculomegaly, simplified gyral patterns, and pontocerebellar hypoplasia [1,4,5,12,34,46,47]. At least 48 deaths were explicitly recorded, ranging from the neonatal period to childhood. Because age at last follow-up was inconsistently reported and ascertainment was retrospective, actuarial survival or genotype-specific survival could not be estimated.

### 3.3. Systematic Evaluation of CSF Asparagine

CSF amino-acid analysis was reported for only a minority of individuals (Supplementary Table S2) [4,5,9,10,11,12,13,45,46,47]. Exact CSF asparagine concentrations were reported for six patients: absent or undetectable in two patients; 1 µmol/L in one patient (reference interval, 1.1–6.9 µmol/L); 2.91 µmol/L, interpreted as normal, in one patient; 4.42 µmol/L in one patient (reference interval, 8–34 µmol/L); and 4.3 µmol/L in one patient (reference interval, 5.9–11.2 µmol/L) [9,11,45,46,47].

Other reports described CSF asparagine only qualitatively, summarized findings for entire cohorts without individual results, or repeated information from secondary sources [4,5,10,12,13,43]. One cohort reported sensitivity of 83% and specificity of 98%, but the index-test definition, diagnostic threshold, individual measurements, and complete contingency table were unavailable for independent verification [4]. The six exact measurements comprise five reduced or undetectable results and one normal result; this selected and very small set cannot be used to calculate sensitivity, specificity, predictive values, or a validated diagnostic threshold.

### 3.4. Population Distribution and Recurrent Variants

Reported patients originated from multiple geographic regions, with relatively large published groups from Saudi Arabia, China, India, Iran, and consanguineous families from the Middle East. Several variants recurred within particular families or regional cohorts, including c.1193A>G (p.Tyr398Cys), c.224A>T (p.Asn75Ile), and c.1424C>A (p.Thr475Asn) in Saudi Arabian families. These observations may reflect founder effects and ascertainment in highly consanguineous populations, but the available literature is not population based. Carrier frequencies, disease prevalence, and population-specific mutation frequencies therefore cannot be estimated reliably from published cases.

## 4. Discussion

Developmental impairment and microcephaly were consistently reported, whereas information on epilepsy, muscle tone, feeding, survival, neuroimaging, and biochemical findings was incomplete in many published cases.

### 4.1. Clinical Phenotype

The patient described herein exhibited the principal features of ASNSD, including developmental and epileptic encephalopathy, progressive microcephaly, profound intellectual disability, spastic tetraparesis, and feeding difficulties requiring gastrostomy. Nevertheless, several aspects of her presentation illustrate the phenotypic variability of this disorder.

Her head circumference at birth was 31 cm (<3rd percentile), consistent with congenital microcephaly, which subsequently progressed markedly. In the original ASNSD series, all nine affected individuals were born with a reduced head circumference and exhibited further postnatal deceleration of head growth [1]. Progressive microcephaly has subsequently been documented in additional reports [34,40], whereas microcephaly developing only after birth has been described less frequently [5]. These observations indicate that impaired brain growth in ASNSD may begin prenatally or become apparent postnatally and may continue to progress throughout childhood. ASNSD should therefore remain in the differential diagnosis of developmental and epileptic encephalopathy even when severe microcephaly is not evident at birth.

Seizures began at 5 months of age, later than the neonatal onset reported in many severely affected patients. Their evolution from infantile spasms to multiple drug-resistant seizure types further illustrates the electroclinical heterogeneity of ASNSD [4,6,11,23,34]. The absence of neonatal seizures should therefore not be considered evidence against the diagnosis.

### 4.2. Progressive Neuroimaging Abnormalities

A notable feature in the reported patient was an initially unremarkable brain MRI followed by severe cortical and subcortical atrophy. Similar radiological evolution has been reported in other individuals [1,4,5,26,34]. An initially normal MRI therefore does not exclude ASNSD; follow-up imaging may be clinically informative when neurological deterioration, deceleration of head growth, or evolving epilepsy provides an independent indication.

The vulnerability of the developing brain may be related to its dependence on local de novo asparagine synthesis. Limited transport of asparagine across the blood–brain barrier increases neuronal dependence on intracellular *ASNS* activity [1,7]. Experimental evidence indicates that asparagine restriction impairs cellular proliferation, providing a plausible mechanism for deficient brain growth and progressive cerebral atrophy [7]. Our findings also demonstrate that an initially normal MRI does not exclude ASNSD and support repeated neuroimaging when the clinical phenotype continues to evolve.

### 4.3. Limitations of Conventional Metabolic Investigations

Routine metabolic investigations were unrevealing in the reported patient, as in other molecularly confirmed cases [4,7,32]. Quantitative plasma amino-acid analysis was performed and showed values within the relevant reference ranges, including a normal asparagine concentration. A normal plasma asparagine concentration therefore does not exclude ASNSD. Across published cases, inconsistent sampling and reporting prevent definition of a characteristic plasma biochemical profile.

Normal routine metabolic testing should therefore not be used to exclude ASNSD. Genomic testing is appropriate in children with otherwise unexplained developmental and epileptic encephalopathy, progressive microcephaly, and cerebral atrophy, after clinically indicated first-line investigations.

### 4.4. Diagnostic Utility of CSF Asparagine

Reduced or undetectable CSF asparagine was initially proposed as a biochemical marker of ASNSD [9]. Following the updated search, exact CSF asparagine concentrations were reported for six patients: five were reduced or undetectable, whereas one was within the relevant reference range [9,11,45,46,47]. The newly identified reports provide additional low values obtained using different local reference intervals but do not resolve the analytical heterogeneity.

Normal or borderline-reduced CSF asparagine concentrations have also been described in molecularly confirmed patients [5,10,11,12,13,43]. One cohort reported sensitivity of 83% and specificity of 98% for CSF biochemical analysis [4], but did not provide an independently reproducible threshold, individual measurements, or a complete two-by-two table. The six reported measurements constitute a small, selected series and cannot substantiate a generally applicable diagnostic-performance claim. Reduced CSF asparagine may support ASNSD, but the literature does not define a validated cut-off or reliable sensitivity, specificity, or predictive values.

CSF asparagine should be interpreted as a supportive biochemical finding rather than a stand-alone diagnostic or exclusionary biomarker. Interpretation may be affected by age, nutritional status, analytical method, reference interval, and sample handling. CSF amino-acid analysis was not performed in the reported patient because parental consent for lumbar puncture was not provided. The case therefore illustrates the value of molecular diagnosis but does not itself contribute biochemical data to the CSF-biomarker analysis. Lumbar puncture should not be undertaken solely to exclude ASNSD when appropriate genomic testing is available.

### 4.5. Novel ASNS Variant and Genotype–Phenotype Considerations

Molecular analysis identified the previously unreported frameshift variant c.81_82del (p.Gly28SerfsTer7) *in trans* with the known missense variant c.97C>T (p.Arg33Cys), which has previously been reported in ASNSD [18]. The novel deletion introduces a premature termination codon in exon 3 and is predicted to result in nonsense-mediated transcript decay or production of a severely truncated protein lacking the essential catalytic domains of asparagine synthetase.

Under the ACMG/AMP framework, c.81_82del was classified as likely pathogenic using PVS1, PM2, and PM3_Moderate. PM3 was applied at Moderate strength in accordance with ClinGen Sequence Variant Interpretation recommendations because the variant was confirmed *in trans* with a previously reported disease-associated variant [15,16,17]. Although the functional impact of this specific variant was not experimentally characterized, previously reported nonsense, frameshift, splice-disrupting, and intragenic deletion variants in ASNSD support loss of ASNS function as an established disease mechanism [1,13,31,35,36,37,38,39]. The molecular findings, segregation pattern, and highly concordant phenotype therefore provide strong evidence that the compound-heterozygous ASNS variants explain the disorder in the reported patient.

Robust genotype–phenotype correlations cannot currently be established. Most variants are private or limited to individual families, published cohorts are small, and follow-up and phenotype definitions are heterogeneous [2,4,6,40]. Recurrent variants in some Saudi Arabian and other consanguineous families may indicate local founder effects, but do not establish population prevalence. A descriptive tendency toward severe disease with biallelic predicted loss-of-function variants remains biologically plausible but unproven and should not be used for individual prognostication.

### 4.6. Therapeutic Considerations

From a pathophysiological perspective, ASNSD belongs to a group of amino acid biosynthesis disorders characterized by deficiency of an enzymatic product rather than accumulation of a toxic substrate. Other disorders involving a similar “product-deficiency” mechanism include glutamine synthetase deficiency and defects of serine biosynthesis. Although these amino acids are classified as non-essential under physiological conditions, they may become conditionally essential when endogenous synthesis is impaired. This provides a rationale for supplementation with the deficient amino acid. However, the prenatal onset of ASNSD and the limited transport of asparagine across the blood–brain barrier may substantially restrict the effectiveness of postnatal supplementation [1,11].

Clinical evidence regarding L-asparagine supplementation remains limited, uncontrolled, and conflicting.

An initial single-patient trial was discontinued after 27 days because of irritability, sleep disturbance, and worsening seizure frequency and severity [48]. Sprute et al. subsequently treated two siblings with 50–100 mg/kg/day for 24 months; treatment was tolerated, but the reported stabilization and mild improvement in one sibling cannot establish efficacy [11]. In a 2026 case, supplementation was started at 5 months of age at 50 mg/kg/day and increased to 100 mg/kg/day. Seizure frequency decreased transiently, but the uncontrolled observation does not establish treatment efficacy [46].

Across these four reported treatment exposures, outcomes were inconsistent and confounded by concomitant antiseizure treatment, age at initiation, advanced prenatal brain involvement, and absence of controlled comparison. No causal therapeutic benefit can be inferred.

There is currently no established disease-modifying treatment for ASNSD. The potential benefits, optimal dosage, timing, long-term safety, and effects on epilepsy remain uncertain.

Earlier intervention might theoretically be more effective, but substantial and potentially irreversible brain injury frequently begins prenatally, and evidence supporting presymptomatic or prenatal treatment is lacking. In our patient, L-asparagine supplementation was not initiated because of her advanced age, profound long-standing neurological impairment and cerebral atrophy, the absence of established efficacy, and the previously reported possibility of seizure exacerbation. Future prospective multicentre studies are required before L-asparagine supplementation can be recommended as standard therapy.

### 4.7. Strengths and Limitations

The principal strength of this study is the integration of a detailed clinical and molecular case description with a systematic synthesis of data from 106 published individuals with ASNSD. Reporting completeness was stated separately for each clinical feature. The CSF analysis distinguishes exact measurements from qualitative descriptions, cohort summaries without individual results, and information derived from secondary sources.

This review is limited by the predominance of case reports and small retrospective series, heterogeneous phenotype definitions, incomplete follow-up, inconsistent biochemical methods and reference intervals, and substantial missing data. No formal risk-of-bias score was applied; the certainty of evidence is inherently very low because the literature consists predominantly of uncontrolled case reports and small case series. Publication and ascertainment bias probably favour severe, early-onset, consanguineous, and diagnostically distinctive cases. Quantitative plasma amino-acid analysis was normal, including the asparagine concentration; quantitative CSF amino-acid analysis was not performed. The novel frameshift variant was not studied functionally. At least 48 reported patients died, but variable and incomplete follow-up precluded survival analysis. Exact CSF asparagine concentrations were available for only six patients; therefore, the evidence cannot support a validated threshold or robust diagnostic-accuracy estimates.

## 5. Conclusions

This study expands the molecular spectrum of ASNSD through identification of the novel likely pathogenic *ASNS* frameshift variant c.81_82del (p.Gly28SerfsTer7), confirmed *in trans* with the previously reported c.97C>T (p.Arg33Cys) variant. The systematic review demonstrates a spectrum ranging from neonatal lethal disease to childhood survival with later-onset epilepsy or slower progression, while also showing that published evidence is strongly affected by incomplete reporting and ascertainment bias.

Normal routine metabolic investigations, an initially unremarkable MRI, or a normal CSF asparagine concentration do not exclude ASNSD. Reduced CSF asparagine may provide supportive evidence, but current data are insufficient to define a validated threshold or reliable diagnostic-accuracy estimates. Molecular testing remains the diagnostic standard in patients with a compatible developmental and epileptic encephalopathy phenotype.

## Figures and Tables

**Figure 1:**
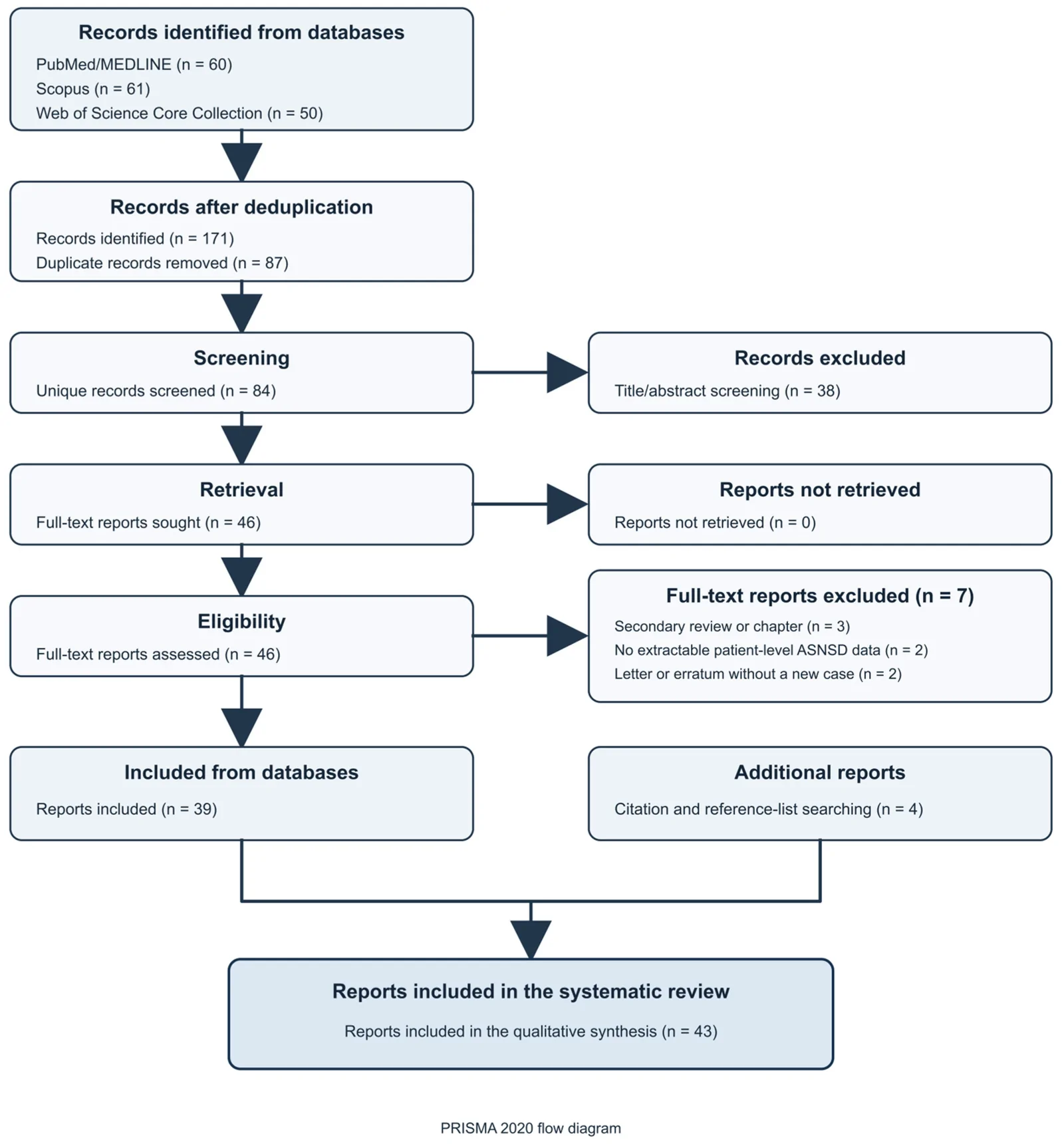
PRISMA 2020 flow diagram of study identification, screening, eligibility assessment, and inclusion.

**Figure 2:**
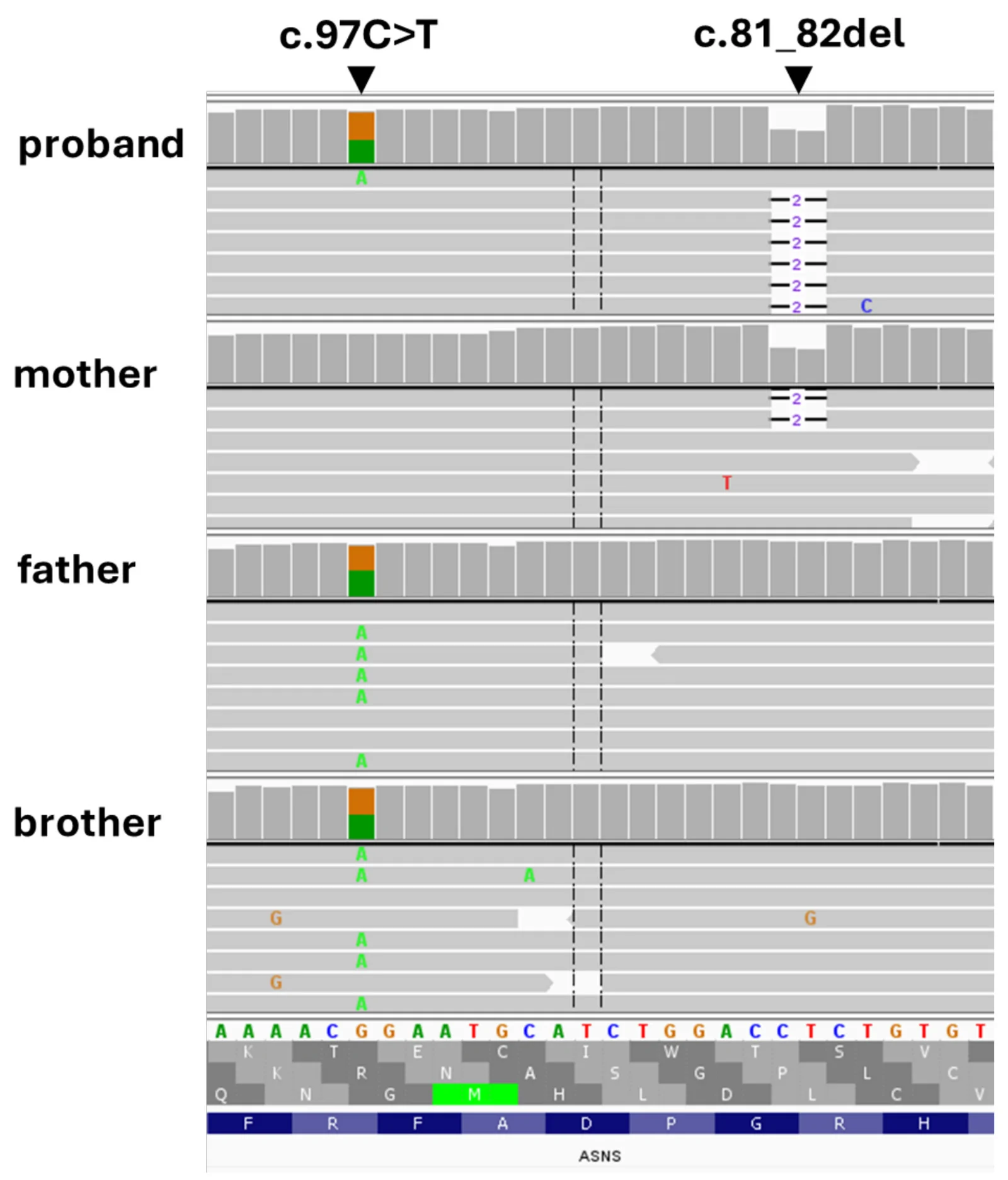
Segregation analysis of the *ASNS* variants. The novel c.81_82del (p.Gly28SerfsTer7) variant was inherited from the mother, whereas c.97C>T (p.Arg33Cys) was inherited from the father. The two variants are therefore *in trans* in the proband, consistent with autosomal recessive inheritance. The healthy brother carried only the paternal c.97C>T variant. Integrative Genomics Viewer images show amplicon-based deep-sequencing results.

**Table 1:** Frequency of selected clinical features among individuals with molecularly confirmed asparagine synthetase deficiency included in the systematic review.

Outcome	Patients with Feature, *n*	Patients with Available Information, *n*	Proportion Among Assessable Patients
Developmental impairment	101	101	100%
Microcephaly	99	106	93%
Epilepsy	72	86	84%
Hypotonia	32	80	40%
Hypertonia/progressive spasticity	23	73	31%
Feeding difficulties	31	57	54%
Abnormal brain MRI	71	73	97%

Note: Information was not available for every clinical feature in every patient. Percentages were calculated only among patients for whom the respective feature could be assessed and should not be interpreted as population-based prevalence estimates.

## Data Availability

The original contributions presented in this study are included in the article and Supplementary Materials. Further inquiries can be directed to the corresponding author.

## Supplementary Materials

The following supporting information can be downloaded at: https://www.mdpi.com/article/10.3390/biom16101377/s1, Table S1: Individual-level clinical and molecular characteristics of 106 published ASNSD cases; Table S2: Systematic summary of reported CSF asparagine measurements; Table S3: Complete database-specific search strategies, record-level screening decisions, deduplication, and full-text exclusions.
