# Supplementary material for: Asparagine Synthetase Deficiency: A Novel ASNS Variant and a Systematic Review of Clinical Phenotypes and Cerebrospinal Fluid Asparagine Levels
Source: Biomolecules. 2026 Sep 22;16(10):1377. doi: 10.3390/biom16101377 (PMC13702534; doi:10.3390/biom16101377)
Supplement: Supplementary file 1 [file biomolecules-16-01377-s001.zip › Table_S1_Individual_clinical_and_molecular_data.pdf]

Table S1. Published individuals with asparagine synthetase deficiency (ASNSD)

Abbreviations: NR, not reported; yr, years; mo, months; wk, weeks; d, days. “Yes” and “No” indicate that a feature was explicitly reported as present or absent. Reference numbers correspond to the manuscript bibliography.

Patient	Country of origin	ASNS genotype	Sex	Age at onset	Follow-up or outcome	Microcephaly	Intellectual disability	Epilepsy	Hypotonia	Hypertonia	Feeding difficulties	Abnormal brain MRI	Reference
1	China	c.1031-6_1041del; c.1049A>G (p.Lys350Arg)	Male	1 mo	6 mo	Yes	Yes	Yes	Yes	No	Yes	Yes	17
2	China	c.1211G>A (p.Arg404His); c.1643C>T (p.Ser548Phe); c.250A>T (p.Met84Leu) [three variants reported; phase not stated]	Female	3 mo	Died at 7 mo	Yes	Yes	Yes	No	No	Yes	NR	17
3	China	c.1211G>A (p.Arg404His); c.1643C>T (p.Ser548Phe); c.250A>T (p.Met84Leu) [three variants reported; phase not stated]	Male	1 mo	Died at 6 mo	No	Yes	Yes	No	No	Yes	NR	17
4	China	c.224A>G (p.Asn75Ser); c.1612A>G (p.Met538Val)	Male	1 yr 4 mo	7 yr	No	Yes	Yes	Yes	No	No	Yes	18
5	China	c.224A>G (p.Asn75Ser); c.1612A>G (p.Met538Val)	Male	1 yr 4 mo	5 yr	No	Yes	Yes	Yes	No	No	Yes	18
6	China	c.1503_1505delAGC; c.776G>C	Female	8 mo	5 yr 3 mo	Yes	Yes	Yes	Yes	Yes	No	NR	19
7	China	c.1341_1343del (p.L448del); c.1283A>G (p.Tyr428Cys)	Male	1 mo	Died at 11 mo	Yes	Yes	Yes	No	Yes	No	Yes	2
8	China	c.1612A>G (p.Met538Val); c.224A>G (p.Asn75Ser)	Male	1 yr 4 mo	5 yr 9 mo	No	Yes	Yes	No	Yes	No	Yes	2
9	China	c.1612A>G (p.Met538Val); c.224A>G (p.Asn75Ser)	Male	1 yr 4 mo	5 yr	No	Yes	Yes	No	Yes	No	NR	2
10	China	c.146G>A (p.Arg49Gln); c.146G>A (p.Arg49Gln)	Male	7 mo	2 yr 4 mo	No	Yes	Yes	No	Yes	No	NR	2
11	China	c.1165_1167del (p.E389del); c.1075G>A (p.Val359Met)	Male	10 mo	5 yr	Yes	Yes	Yes	No	Yes	No	Yes	2
12	China	c.1424C>T (p.Thr475Ile); c.1649G>A (p.Arg550His)	Male	1 mo	Died at 5 mo	Yes	Yes	Yes	No	Yes	No	Yes	2
13	China	c.666_667delCT (p.Leu222PhefsTer5); c.1193A>C (p.Tyr398Ser)	Male	1 mo	Died at 3 mo	Yes	Yes	Yes	No	Yes	Yes	Yes	20
14	China	c.1612A>G (p.Met538Val); c.224A>G (p.Asn75Ser)	Male	1 yr 3 mo	8 yr	No	Yes	Yes	No	No	Yes	Yes	21
15	China	c.1612A>G (p.Met538Val); c.224A>G (p.Asn75Ser)	Male	1 yr	5 yr 10 mo	Yes	Yes	Yes	No	No	No	NR	21
16	China	c.146G>A; c.86C>A	Female	4 mo	2 yr 2 mo	Yes	Yes	Yes	No	Yes	Yes	NR	22
17	China	c.4T>G (p.Cys2Gly) (homozygous)	Female	3 mo	Died at 6 mo	Yes	Yes	Yes	Yes	No	Yes	Yes	23
18	China	c.368T>C (p.Phe123Ser); c.1649G>A (p.Arg550His)	Female	2 mo	3 mo	Yes	Yes	Yes	No	Yes	Yes	Yes	24
19	China	c.1424C>T (p.Thr475Ile); c.666_667delCT (p.Leu222PhefsTer5)	Male	2 mo	5 mo	Yes	Yes	Yes	No	No	No	Yes	25
20	China	c.551C>T; c.944A>C	Male	Toddlerhood	10 yr	Yes	Yes	NR	NR	NR	NR	NR	26
21	China	c.551C>T; c.944A>C	Female	Infancy	Died at 6 yr	Yes	Yes	NR	NR	NR	NR	NR	26
22	China	c.97C>T (p.Arg33Cys); c.1031-2_1033del	NR	Fetus	NR	Yes	NR	NR	NR	NR	NR	NR	27
23	Iran	c.1084T>G (p.Phe362Val) (homozygous)	Male	1 mo	14 yr	Yes	Yes	Yes	No	NR	NR	Yes	1
24	Iran	c.1084T>G (p.Phe362Val) (homozygous)	Male	1 mo	14 yr	Yes	Yes	Yes	No	NR	NR	Yes	1
25	Iran	c.1084T>G (p.Phe362Val) (homozygous)	Male	1 mo	12 yr	Yes	Yes	Yes	No	NR	NR	Yes	1
26	Iran	c.1084T>G (p.Phe362Val) (homozygous)	Male	Birth	4 mo	Yes	Yes	No	No	NR	NR	Yes	1
27	Iran	c.538T>A (homozygous)	Female	10 d	11 yr	Yes	Yes	Yes	No	No	No	Yes	1

Supplementary Table S1. Continued — patients 28–54

Patient	Country of origin	ASNS genotype	Sex	Age at onset	Follow-up or outcome	Microcephaly	Intellectual disability	Epilepsy	Hypotonia	Hypertonia	Feeding difficulties	Abnormal brain MRI	Reference
28	Bangladesh	c.1648C>T (p.Arg550Cys) (homozygous)	Male	Birth	3 mo	Yes	Yes	No	Yes	NR	NR	Yes	10
29	Bangladesh	c.1648C>T (p.Arg550Cys) (homozygous)	Female	Birth	6 mo	Yes	Yes	No	Yes	NR	NR	Yes	10
30	Canada	c.1648C>T (p.Arg550Cys); c.17C>A (p.Ala6Glu)	Male	4 d	9 d	Yes	Yes	Yes	Yes	NR	NR	Yes	1
31	Canada	c.1648C>T (p.Arg550Cys); c.17C>A (p.Ala6Glu)	Male	9 mo	11 mo	Yes	Yes	Yes	Yes	NR	NR	Yes	1
32	Canada	c.1648C>T (p.Arg550Cys); c.17C>A (p.Ala6Glu)	Male	8 d	1 yr	Yes	Yes	Yes	Yes	NR	NR	Yes	1
33	Turkey	c.1108C>T (p.Leu370Phe) (homozygous)	Male	2 mo	5 yr 9 mo	Yes	Yes	Yes	Yes	No	No	Yes	11
34	Turkey	c.1108C>T (p.Leu370Phe) (homozygous)	Male	6 mo	3 yr 9 mo	Yes	Yes	Yes	Yes	No	No	Yes	11
35	Turkey	c.83G>A (p.Arg28Gln); c.1082A>T (p.Glu361Val)	Male	6 mo	7 yr	Yes	Yes	Yes	Yes	No	No	Yes	28
36	India	c.788C>T (p.Ser263Phe) (homozygous)	Male	5 d	Died at 1 mo	Yes	Yes	Yes	No	No	Yes	Yes	3
37	India	c.788C>T (p.Ser263Phe) (homozygous)	Female	5 d	Died at 5 d	Yes	Yes	No	No	Yes	Yes	Yes	3
38	India	c.146G>A (p.Arg49Gln) (homozygous)	Male	Birth	3 yr	Yes	Yes	Yes	No	No	No	NR	3
39	India	c.1211G>A (p.Arg404His) (homozygous)	Male	4 d	Died at 10 d	Yes	Yes	Yes	No	Yes	Yes	Yes	29
40	India	c.224A>G (p.Asn75Ser); c.413A>C (p.Asp138Ala)	Female	Neonatal period	Died in early infancy	Yes	Yes	No	No	No	No	Yes	29
41	India	c.1649G>A (p.Arg550His) (homozygous)	Male	Birth	Died at 1 yr 1 mo	Yes	Yes	Yes	No	No	No	Yes	29
42	India	c.1019G>A (p.Arg340His) (homozygous)	Female	Birth	Died at 6 mo	Yes	Yes	No	Yes	Yes	Yes	Yes	30
43	India	c.1019G>A (p.Arg340His) (homozygous)	Female	Birth	Died at 11 d	Yes	Yes	No	Yes	Yes	Yes	Yes	30
44	India	c.1138G>T (p.Ala380Ser) (homozygous)	Female	3 mo	2 yr 6 mo	Yes	Yes	Yes	No	Yes	Yes	Yes	31
45	Saudi Arabia	c.1193A>G (p.Tyr398Cys) (homozygous)	Male	NR	Died at 6 yr	Yes	Yes	Yes	Yes	No	NR	Yes	4
46	Saudi Arabia	c.1193A>G (p.Tyr398Cys) (homozygous)	Female	NR	3 mo	Yes	Yes	NR	NR	NR	NR	Yes	4
47	Saudi Arabia	c.1193A>G (p.Tyr398Cys) (homozygous)	Male	NR	Died at 1 yr 8 mo	Yes	Yes	Yes	Yes	No	NR	NR	4
48	Saudi Arabia	c.1193A>G (p.Tyr398Cys) (homozygous)	Male	NR	Died (details unknown)	Yes	Yes	NR	NR	NR	NR	NR	4
49	Saudi Arabia	c.1193A>G (p.Tyr398Cys) (homozygous)	Male	NR	Died (details unknown)	Yes	Yes	NR	NR	NR	NR	NR	4
50	Saudi Arabia	c.1193A>G (p.Tyr398Cys) (homozygous)	Female	NR	Died (details unknown)	Yes	Yes	NR	NR	NR	NR	NR	4
51	Saudi Arabia	c.1137+1G>A (homozygous)	Male	NR	Died at 3 yr 7 mo	Yes	Yes	Yes	No	No	NR	Yes	4
52	Saudi Arabia	c.1137+1G>A (homozygous)	Male	NR	Died at 3 mo	Yes	Yes	No	No	No	NR	NR	4
53	Saudi Arabia	c.1211G>A (p.Arg404His) (homozygous)	Male	NR	Died at 22 d	Yes	Yes	Yes	Yes	No	NR	Yes	4
54	Saudi Arabia	c.1211G>A (p.Arg404His) (homozygous)	Female	NR	Died at 4 mo	Yes	Yes	NR	NR	NR	NR	NR	4

Supplementary Table S1. Continued — patients 55–81

Patient	Country of origin	ASNS genotype	Sex	Age at onset	Follow-up or outcome	Microcephaly	Intellectual disability	Epilepsy	Hypotonia	Hypertonia	Feeding difficulties	Abnormal brain MRI	Reference
55	Saudi Arabia	c.1211G>A (p.Arg404His) (homozygous)	Female	NR	Died at 4 mo	Yes	Yes	NR	NR	NR	NR	NR	4
56	Saudi Arabia	c.1193A>G (p.Tyr398Cys) (homozygous)	Male	NR	Died at 2 yr	Yes	Yes	NR	NR	NR	NR	NR	4
57	Saudi Arabia	c.1193A>G (p.Tyr398Cys) (homozygous)	Female	NR	Died at 4 yr	Yes	Yes	No	Yes	No	NR	Yes	4
58	Saudi Arabia	c.1193A>G (p.Tyr398Cys) (homozygous)	Female	NR	Died at 3 yr 6 mo	Yes	Yes	Yes	NR	NR	NR	NR	4
59	Saudi Arabia	c.1193A>G (p.Tyr398Cys) (homozygous)	Male	NR	1 yr 7 mo	Yes	Yes	No	Yes	No	NR	Yes	4
60	Saudi Arabia	c.224A>T (p.Asn75Ile) (homozygous)	Male	NR	Died at 3 yr	Yes	Yes	Yes	No	No	No	NR	4
61	Saudi Arabia	c.224A>T (p.Asn75Ile) (homozygous)	Female	NR	Died at 2 yr 7 mo	Yes	Yes	No	No	No	No	Yes	4
62	Saudi Arabia	c.224A>T (p.Asn75Ile) (homozygous)	Male	NR	3 yr	Yes	Yes	Yes	No	No	NR	Yes	4
63	Saudi Arabia	c.224A>T (p.Asn75Ile) (homozygous)	Male	NR	Died in toddlerhood	Yes	Yes	NR	NR	NR	NR	NR	4
64	Saudi Arabia	c.224A>T (p.Asn75Ile) (homozygous)	Male	NR	Died in toddlerhood	Yes	Yes	NR	NR	NR	NR	NR	4
65	Saudi Arabia	c.224A>T (p.Asn75Ile) (homozygous)	Male	NR	Died in toddlerhood	Yes	Yes	NR	NR	NR	NR	NR	4
66	Saudi Arabia	c.224A>T (p.Asn75Ile) (homozygous)	Male	NR	Died in toddlerhood	Yes	Yes	NR	NR	NR	NR	NR	4
67	Saudi Arabia	c.224A>T (p.Asn75Ile) (homozygous)	Female	NR	2 yr 7 mo	Yes	Yes	Yes	No	No	NR	Yes	4
68	Saudi Arabia	c.1424C>A (p.Thr475Asn) (homozygous)	Male	NR	2 yr 2 mo	Yes	Yes	Yes	Yes	No	NR	Yes	4
69	Saudi Arabia	c.1424C>A (p.Thr475Asn) (homozygous)	Male	NR	5 years	Yes	Yes	Yes	NR	NR	NR	NR	4
70	Saudi Arabia	c.1424C>A (p.Thr475Asn) (homozygous)	Female	NR	2 yr 10 mo	Yes	Yes	Yes	Yes	No	NR	Yes	4
71	Saudi Arabia	c.1424C>A (p.Thr475Asn) (homozygous)	Female	NR	7 years	Yes	Yes	NR	Yes	No	NR	Yes	4
72	Saudi Arabia	c.1424C>A (p.Thr475Asn) (homozygous)	Male	NR	NR	Yes	Yes	NR	NR	NR	NR	NR	4
73	Saudi Arabia	c.674-1G>A (homozygous)	Male	NR	Died at 3 months	Yes	Yes	Yes	Yes	No	NR	Yes	4
74	Saudi Arabia	c.674-1G>A (homozygous)	Male	NR	Died at 3 months	Yes	Yes	NR	NR	NR	NR	NR	4
75	Saudi Arabia	c.1219C>T (p.Arg407Ter) (homozygous)	Female	NR	Died at 8 d	Yes	Yes	No	No	No	NR	No	4
76	Saudi Arabia	c.1160A>G (p.Tyr377Cys) (homozygous)	Male	NR	4 years	Yes	Yes	Yes	Yes	No	Yes	Yes	9
77	Saudi Arabia	c.1160A>G (p.Tyr377Cys) (homozygous)	Female	NR	2 years	Yes	Yes	Yes	Yes	No	Yes	Yes	9
78	Saudi Arabia	c.970C>T (p.Arg324Ter) (homozygous)	Male	Birth	Died at 6 wk	Yes	Yes	Yes	No	Yes	Yes	Yes	12
79	Saudi Arabia	c.944A>G (p.Tyr315Cys) (homozygous)	Male	3 wk	4 years	Yes	Yes	Yes	No	Yes	No	Yes	12
80	United Arab Emirates	c.1193A>C (p.Tyr398Cys) (homozygous)	Male	Birth	5 years	Yes	Yes	Yes	No	Yes	NR	Yes	32
81	United Arab Emirates	c.146G>A (p.Arg49Gln) (homozygous)	Male	6 months	7 years	Yes	Yes	Yes	No	No	Yes	Yes	13

Supplementary Table S1. Continued — patients 82–106

Patient	Country of origin	ASNS genotype	Sex	Age at onset	Follow-up or outcome	Microcephaly	Intellectual disability	Epilepsy	Hypotonia	Hypertonia	Feeding difficulties	Abnormal brain MRI	Reference
82	United Arab Emirates	c.146G>A (p.Arg49Gln) (homozygous)	Female	6 months	2 yr 6 mo	Yes	Yes	Yes	Yes	No	No	No	13
83	France	c.1439C>T (p.Ser480Phe); c.1648C>T (p.Arg550Cys)	Male	Birth	8 months	Yes	Yes	Yes	No	No	Yes	Yes	33
84	France	c.1439C>T (p.Ser480Phe); c.1648C>T (p.Arg550Cys)	Female	Birth	8 months	Yes	Yes	Yes	No	No	No	Yes	33
85	France	c.144C>A (p.His48Gln) (homozygous)	Female	8 months	4 years	Yes	Yes	Yes	Yes	No	No	Yes	34
86	Australia	c.866G>C (p.Gly289Ala); c.1010C>T (p.Thr337Ile)	Male	5 d	8 yr	Yes	Yes	Yes	Yes	No	Yes	Yes	7
87	Australia	c.1476+1G>A (homozygous)	Male	Birth	Died at 2 mo	Yes	Yes	No	NR	NR	NR	Yes	35
88	Australia	c.1476+1G>A (homozygous)	Male	Birth	Died at 1 mo	Yes	Yes	No	NR	NR	NR	Yes	35
89	Japan	c.434T>C (p.Leu145Ser); c.740T>G (p.Leu247Trp)	Male	3 mo	2 yr 2 mo	Yes	Yes	Yes	No	Yes	Yes	Yes	5
90	Japan	c.1466T>A (p.Val489Asp); c.1623_1624del (p.Trp541CysfsTer5)	Male	Birth	8 yr	Yes	Yes	Yes	Yes	Yes	Yes	Yes	5
91	United States of America	c.1097G>A; c.728T>C	NR	NR	Died at 1 yr 5 mo	Yes	Yes	Yes	No	Yes	Yes	Yes	36
92	United States of America	c.479delC (p.Glu160LeufsTer8); c.1282T>G (p.Tyr428Cys)	Male	NR	Died at 2 yr	Yes	Yes	Yes	No	No	Yes	Yes	8
93	United States of America	c.614A>C (p.His205Pro); c.1192dupT (p.Tyr398LeufsTer4)	Male	11 months	4 years	Yes	Yes	Yes	Yes	No	No	Yes	37
94	United States of America	c.1118G>T (p.Gly373Val); c.1556G>A (p.Arg519His)	Female	1 yr 1 mo	9 yr	Yes	Yes	Yes	No	No	No	NR	38
95	Germany	c.1165G>C (p.Glu389Gln); c.601delA (p.Met201TrpfsTer28)	Female	Birth	19 yr	Yes	Yes	Yes	No	No	Yes	Yes	39
96	Germany	c.1165G>C (p.Glu389Gln); c.601delA (p.Met201TrpfsTer28)	Female	Birth	16 yr	Yes	Yes	Yes	No	No	Yes	Yes	39
97	Egypt	c.397_398GT>CA (p.Val133Gln) (homozygous)	Female	Birth	Died at 10 d	Yes	Yes	Yes	No	No	Yes	Yes	40
98	Egypt	c.397_398GT>CA (p.Val133Gln) (homozygous)	Female	Birth	Died at 8 mo	Yes	Yes	Yes	No	No	Yes	Yes	40
99	Italy	c.761G>T (p.Gly254Val) (homozygous)	Male	Birth	10 mo	Yes	Yes	Yes	Yes	No	Yes	Yes	41
100	South Korea/Vietnam	c.1018C>T (p.Arg340Cys); c.1555C>T (p.Arg519Cys)	Male	4 mo	1 yr 7 mo	Yes	Yes	Yes	No	No	Yes	Yes	6
101	Oman	c.776-5T>G; homozygous	NR	Birth	Died in early infancy	Yes	NR	NR	NR	NR	NR	NR	45
102	Oman	c.776-5T>G; homozygous	NR	Birth	Died in early infancy	Yes	NR	NR	NR	NR	NR	NR	45
103	Oman	c.776-5T>G; homozygous	NR	Birth	Died in early infancy	Yes	NR	NR	NR	NR	NR	NR	45
104	Oman	c.776-5T>G; homozygous	Male	Birth	Died in early infancy	Yes	NR	Yes	NR	Yes	NR	Yes	45
105	Türkiye	Homozygous ASNS splice-site variant; exact HGVS not reported in accessible abstract	Female	Early infancy	NR	Yes	Yes	Yes	NR	NR	NR	Yes	46
106	Croatia	c.904-1G>A; homozygous	NR	Birth	Died at 6 mo	Yes	Yes	Yes	NR	NR	NR	Yes	47
