# Supplementary material for: Asparagine Synthetase Deficiency: A Novel ASNS Variant and a Systematic Review of Clinical Phenotypes and Cerebrospinal Fluid Asparagine Levels
Source: Biomolecules. 2026 Sep 22;16(10):1377. doi: 10.3390/biom16101377 (PMC13702534; doi:10.3390/biom16101377)
Supplement: Supplementary file 1 [file biomolecules-16-01377-s001.zip › Table_S2_CSF_asparagine.pdf]

Table S2. Cerebrospinal fluid asparagine measurements in published individuals with ASNSD

Abbreviations: ASNSD, asparagine synthetase deficiency; CSF, cerebrospinal fluid; NR, not reported or not extractable. Reference numbers correspond to the manuscript bibliography.

Reference	Reporting status	Low/undetectable, n	Normal/borderline, n	CSF asparagine result or evidence note
10	Reported	0	1	CSF amino acids investigated; CSF asparagine within normal range according to accessible snippets/GeneReviews.
11	Reported	0	1	Index patient 1: CSF asparagine 2.91 µmol/L; patient 2 not reported.
4	Reported at cohort level	Majority	Some	Cohort-level CSF amino-acid analysis: low CSF asparagine in the majority, but not all, patients; reported sensitivity 83% and specificity 98%. Individual values were unavailable.
9	Reported	2	0	Patient 1: not detected; Patient 2: 1 µmol/L; local CSF reference range 1.1–6.9 µmol/L.
12	Reported	0	2	Both patients had normal plasma and CSF asparagine; exact values not extracted from accessible table text.
13	Reported	0	1	Sprute review cites Sacharow among cases with normal/borderline CSF asparagine; exact value not available in accessible text.
5	Reported	1	1	GeneReviews cites Yamamoto for low/not detected CSF asparagine; Sprute review indicates Yamamoto included both non-detectable and normal/borderline results. Exact assignment unavailable from accessible text.
45	Reported	1	0	One tested Omani child had absent CSF asparagine; plasma asparagine was also reduced. Exact numerical concentration and local reference interval were not reported.
46	Reported	1	0	CSF asparagine 4.42 µmol/L; local reference interval 8–34 µmol/L.
47	Reported	1	0	CSF asparagine 4.3 µmol/L; local reference interval 5.9–11.2 µmol/L.
