# Supplementary material for: Asparagine Synthetase Deficiency: A Novel ASNS Variant and a Systematic Review of Clinical Phenotypes and Cerebrospinal Fluid Asparagine Levels
Source: Biomolecules. 2026 Sep 22;16(10):1377. doi: 10.3390/biom16101377 (PMC13702534; doi:10.3390/biom16101377)
Supplement: Supplementary file 1 [file biomolecules-16-01377-s001.zip › Table_S3_Search_strategies_and_screening.pdf]

Table S3 Search strategies and study selection

Search date: 20 September 2026. Searches were conducted from database inception without language restrictions.

Database	Search strategy	Date searched	Records
PubMed/MEDLINE	("asparagine synthetase deficiency"[Title/Abstract] OR "ASNS deficiency"[Title/Abstract] OR ASNSD[Title/Abstract] OR (ASNS[Title/Abstract] AND (microcephaly[Title/Abstract] OR "epileptic encephalopathy"[Title/Abstract] OR "developmental delay"[Title/Abstract] OR "developmental impairment"[Title/Abstract]))) AND ("1900/01/01"[Date - Publication] : "2026/09/20"[Date - Publication])	20 Sep 2026	60
Scopus	TITLE-ABS-KEY("asparagine synthetase deficiency" OR "ASNS deficiency" OR ASNSD OR (ASNS AND (microcephaly OR "epileptic encephalopathy" OR "developmental delay" OR "developmental impairment"))) AND PUBYEAR < 2027	20 Sep 2026	61
Web of Science Core Collection	TS=("asparagine synthetase deficiency" OR "ASNS deficiency" OR ASNSD OR (ASNS AND (microcephaly OR "epileptic encephalopathy" OR "developmental delay" OR "developmental impairment")))	20 Sep 2026	50

PRISMA accounting

- Database records identified: 171
- Duplicate records removed: 87
- Unique records screened: 84
- Records excluded after title/abstract screening: 38
- Full-text reports assessed: 46
- Full-text reports excluded: 7

- Database-indexed reports included: 39
- Additional reports identified by citation/reference searching: 4
- Reports included in qualitative synthesis: 43

Full-text exclusions

Report	Reason
Lomelino et al., 2017; doi:10.1074/jbc.R117.819060	Secondary review; no new individual data
Antenatal manifestations of ASNSD; doi:10.1007/s10545-016-9937-x	No new molecularly confirmed individual ASNSD data
Rare Genomes Project; doi:10.1186/s40246-024-00604-w	No extractable clinical information for an individual with ASNSD for this review
Letter to the editor; doi:10.1016/j.wneu.2019.05.081	No new patient
Erratum; doi:10.1007/s11011-017-0102-5	No new patient
Asparagine Synthetase Deficiency. GeneReviews	Secondary review; no new individual data
Disorders of glutamine, serine and asparagine metabolism	Book chapter; no new individual data

Record-level screening log

No.	Source(s)	Title	DOI	Decision and reason
1	Web of Science	A MILDER PHENOTYPE OF ASPARAGINE SYNTHETASE DEFICIENCY AND INITAL RESPONSE TO ASPARAGINE AND GLUTAMINE SUPPLEMENTATION	Not reported	Excluded at title/abstract: Not an eligible molecularly confirmed ASNSD clinical report
2	Web of Science	A Neuro-metabolic Syndrome that Needs to Be Discovered: A Child with Late Onset Asparagine Synthetase Deficiency	10.1055/s-0041-1739488	Excluded at title/abstract: Not an eligible molecularly confirmed ASNSD clinical report
3	PubMed/MEDLINE; Scopus; Web of Science	A method for measurement of human asparagine synthetase (ASNS) activity and application to ASNS protein variants associated with ASNS deficiency	10.1093/biomethods/bpad026	Excluded at title/abstract: Not an eligible molecularly confirmed ASNSD clinical report
4	PubMed/MEDLINE; Scopus; Web of Science	Analysis of Enzyme Activity and Cellular Function for the N80S and S480F Asparagine Synthetase Variants Expressed in a Child with Asparagine Synthetase Deficiency	10.3390/ijms24010559	Excluded at title/abstract: Not an eligible molecularly confirmed ASNSD clinical report

5	Scopus	Analysis of a family with asparagine synthase deficiency caused by ASNS gene mutation and literature review; [ASNS 基因变异致天冬酰胺合成酶缺乏症一家系分析并文献复习]	10.3760/cma.j.cn101070-20200415-00645	Excluded at title/abstract: Not an eligible molecularly confirmed ASNSD clinical report
6	PubMed/MEDLINE; Scopus	Asparagine Synthetase Marks a Distinct Dependency Threshold for Cardiomyocyte Dedifferentiation	10.1161/circulationaha.123.063965	Excluded at title/abstract: Not an eligible molecularly confirmed ASNSD clinical report
7	Web of Science	Asparagine as a signal for glutamine sufficiency via asparagine synthetase: a fresh evidence-based framework in physiology and oncology	10.1152/ajpcell.00316.2024	Excluded at title/abstract: Not an eligible molecularly confirmed ASNSD clinical report
8	Web of Science	Asparagine synthetase promoter hypermethylation is required, but not sufficient, for sensitivity to Asparlas in patient-derived models of hepatocellular carcinoma	10.1016/j.ejphar.2025.178241	Excluded at title/abstract: Not an eligible molecularly confirmed ASNSD clinical report
9	Web of Science	Asparagine synthetase regulates the proliferation and differentiation of chicken skeletal muscle satellite cells	10.5713/ab.24.0271	Excluded at title/abstract: Not an eligible molecularly confirmed ASNSD clinical report
10	PubMed/MEDLINE; Scopus	Aspartic Acid in Health and Disease	10.3390/nu15184023	Excluded at title/abstract: Not an eligible molecularly confirmed ASNSD clinical report
11	Web of Science	Association of allele-specific methylation of the ASNS gene with asparaginase sensitivity and prognosis in T-ALL	10.1182/bloodadvances.2021004271	Excluded at title/abstract: Not an eligible molecularly confirmed ASNSD clinical report
12	PubMed/MEDLINE	Conformational Remodeling Underlies Activity Loss in Disease-Linked Asparagine Synthetase Variant	10.64898/2026.02.01.703106	Excluded at title/abstract: Not an eligible molecularly confirmed ASNSD clinical report
13	PubMed/MEDLINE; Scopus; Web of Science	Congenital microcephaly with early onset epileptic encephalopathy caused by ASNS gene mutation: A case report	10.1097/md.0000000000020507	Excluded at title/abstract: Not an eligible molecularly confirmed ASNSD clinical report
14	PubMed/MEDLINE; Scopus	DOT1L activity affects neural stem cell division mode and reduces	10.15252/embr.202256233	Excluded at title/abstract: Not an eligible molecularly

		differentiation and ASNS expression		confirmed ASNSD clinical report
15	PubMed/MEDLINE; Scopus	Deficiency in asparagine synthetase expression in rectal cancers receiving concurrent chemoradiotherapy: negative prognostic impact and therapeutic relevance	10.1007/s13277-014-1895-z	Excluded at title/abstract: Not an eligible molecularly confirmed ASNSD clinical report
16	Scopus	Diagnostic power and clinical impact of exome sequencing in a cohort of 500 patients with rare diseases	10.1002/ajmg.c.31860	Excluded at title/abstract: Not an eligible molecularly confirmed ASNSD clinical report
17	Scopus	Diagnostic yield of targeted massively parallel sequencing in children with epileptic encephalopathy	10.1016/j.seizure.2018.05.005	Excluded at title/abstract: Not an eligible molecularly confirmed ASNSD clinical report
18	Web of Science	Direct pharmacological targeting of asparagine synthetase to overcome resistance to L-asparaginase in ALL therapy	10.1172/jci.insight.203777	Excluded at title/abstract: Not an eligible molecularly confirmed ASNSD clinical report
19	PubMed/MEDLINE; Scopus	Effects of A6E Mutation on Protein Expression and Supramolecular Assembly of Yeast Asparagine Synthetase	10.3390/biology10040294	Excluded at title/abstract: Not an eligible molecularly confirmed ASNSD clinical report
20	Web of Science	Expanding the clinical spectrum of asparagine synthetase deficiency	10.1016/j.gim.2022.01.058	Excluded at title/abstract: Not an eligible molecularly confirmed ASNSD clinical report
21	PubMed/MEDLINE; Scopus	From ALL to Myeloid and NK Malignancies: Operationalizing "ASNS-Low" for L-Asparaginase Repurposing and Combination Therapy	10.3390/biom16060792	Excluded at title/abstract: Not an eligible molecularly confirmed ASNSD clinical report
22	Web of Science	Genome-wide identification of asparagine synthetase genes (CsASNs) and their potential roles in nitrogen remobilization from mature leaves to new shoots of tea plants (Camellia sinensis L.)	10.1016/j.indcrop.2025.122356	Excluded at title/abstract: Not an eligible molecularly confirmed ASNSD clinical report
23	PubMed/MEDLINE; Scopus; Web of Science	In vitro functional analysis of four variants of human asparagine synthetase	10.1002/jimd.12408	Excluded at title/abstract: Not an eligible molecularly confirmed ASNSD clinical report
24	Web of Science	METTL1-mediated m7G modification of ASNS mRNA regulates asparagine metabolism	10.1038/s41388-026-03806-9	Excluded at title/abstract: Not an eligible molecularly

		reprogramming to promote hepatocellular carcinoma progression		confirmed ASNSD clinical report
25	PubMed/MEDLINE; Scopus; Web of Science	Metabolomic Profiling of Asparagine Deprivation in Asparagine Synthetase Deficiency Patient-Derived Cells	10.3390/nu15081938	Excluded at title/abstract: Not an eligible molecularly confirmed ASNSD clinical report
26	Scopus	Microcephaly in Neurometabolic Diseases	10.3390/children9010097	Excluded at title/abstract: Not an eligible molecularly confirmed ASNSD clinical report
27	Web of Science	NUCKS1, a LINC00629-upregulated gene, facilitated osteosarcoma progression and metastasis by elevating asparagine synthesis	10.1038/s41419-023-06010-9	Excluded at title/abstract: Not an eligible molecularly confirmed ASNSD clinical report
28	PubMed/MEDLINE; Scopus	Pancreatic tumor sensitivity to plasma L-asparagine starvation	10.1097/mpa.0b013e318247d903	Excluded at title/abstract: Not an eligible molecularly confirmed ASNSD clinical report
29	Web of Science	Pathophysiological consequences of microcephaly-associated mutations in the asparagine synthetase (ASNS) gene	Not reported	Excluded at title/abstract: Not an eligible molecularly confirmed ASNSD clinical report
30	Scopus	Prenatal diagnosis of microcephaly through combined MRI and ultrasonography: Analysis of a case series	10.1097/md.0000000000036623	Excluded at title/abstract: Not an eligible molecularly confirmed ASNSD clinical report
31	Web of Science	Primary cilia sense glutamine availability and respond via asparagine synthetase	10.1038/s42255-023-00754-6	Excluded at title/abstract: Not an eligible molecularly confirmed ASNSD clinical report
32	Web of Science	Promoter demethylation of the asparagine synthetase gene is required for ATF4-dependent adaptation to asparagine depletion	10.1074/jbc.ra119.010447	Excluded at title/abstract: Not an eligible molecularly confirmed ASNSD clinical report
33	Web of Science	Reverse Phase Proteomic Array Profiling of Asparagine Synthetase Expression in Newly Diagnosed Acute Myeloid Leukemia	10.1021/acs.jproteome.4c00130	Excluded at title/abstract: Not an eligible molecularly confirmed ASNSD clinical report
34	Scopus	Stochastic optimal design of adjacent structures with negative stiffness damper under non-stationary seismic excitation considering soil conditions	10.1016/j.istruc.2025.109910	Excluded at title/abstract: Not an eligible molecularly confirmed ASNSD clinical report

35	Scopus	Targeting amino acid metabolism in cancer	10.1016/bs.ircmb.2022.08.001	Excluded at title/abstract: Not an eligible molecularly confirmed ASNSD clinical report
36	PubMed/MEDLINE; Scopus	The role of asparagine synthetase on nutrient metabolism in pancreatic disease	10.1016/j.pan.2020.08.002	Excluded at title/abstract: Not an eligible molecularly confirmed ASNSD clinical report
37	Web of Science	Utility of ASNS gene methylation evaluated with the HPLC method as a pharmacogenomic biomarker to predict asparaginase sensitivity in BCP-ALL	10.1080/15592294.2023.2268814	Excluded at title/abstract: Not an eligible molecularly confirmed ASNSD clinical report
38	PubMed/MEDLINE	Utility of AlphaMissense predictions in Asparagine Synthetase deficiency variant classification	10.1101/2023.10.30.564808	Excluded at title/abstract: Not an eligible molecularly confirmed ASNSD clinical report
39	PubMed/MEDLINE; Scopus	Antenatal manifestations of inborn errors of metabolism: autopsy findings suggestive of a metabolic disorder	10.1007/s10545-016-9937-x	Full text excluded: No new molecularly confirmed individual ASNSD data
40	PubMed/MEDLINE	Asparagine Synthetase Deficiency	Not reported	Full text excluded: Secondary review or book chapter; no new individual data
41	PubMed/MEDLINE; Scopus	Asparagine synthetase: Function, structure, and role in disease	10.1074/jbc.r117.819060	Full text excluded: Secondary review; no new individual data
42	PubMed/MEDLINE; Scopus	Critical assessment of variant prioritization methods for rare disease diagnosis within the rare genomes project	10.1186/s40246-024-00604-w	Full text excluded: No extractable clinical information for an individual with ASNSD for this review
43	Scopus	Disorders of glutamine, serine and asparagine metabolism	10.1007/978-3-662-63123-2_24	Full text excluded: Secondary review or book chapter; no new individual data
44	PubMed/MEDLINE; Scopus	Erratum to: Asparagine synthetase deficiency-report of a novel mutation and review of literature	10.1007/s11011-017-0102-5	Full text excluded: Erratum without a new patient
45	Scopus	Letter to the Editor Regarding “Cyst-Peritoneal Shunt for the Treatment of a Progressive Intracerebral Cyst Associated with ASNS Mutation: Case Report and Literature Review”	10.1016/j.wneu.2019.05.081	Full text excluded: Letter without a new patient
46	PubMed/MEDLINE; Scopus; Web of Science	A missense variant in Exon 9 of the ASNS gene causes splicing	10.3389/fgene.2026.1799796	Included: Molecularly confirmed ASNSD clinical report

		abnormality in an Infant with asparagine synthetase deficiency		
47	PubMed/MEDLINE; Scopus; Web of Science	A novel compound heterozygous missense mutation in ASNS broadens the spectrum of asparagine synthetase deficiency	10.1002/mgg3.1235	Included: Molecularly confirmed ASNSD clinical report
48	PubMed/MEDLINE; Scopus; Web of Science	A novel variant in ASNS gene responsible for syndromic intellectual disability and microcephaly: Case report and literature review	10.1002/mgg3.2424	Included: Molecularly confirmed ASNSD clinical report
49	PubMed/MEDLINE; Scopus; Web of Science	An intractable epilepsy phenotype of ASNS novel mutation in two patients with asparagine synthetase deficiency	10.1016/j.cca.2022.04.989	Included: Molecularly confirmed ASNSD clinical report
50	PubMed/MEDLINE; Scopus; Web of Science	Asparagine Synthetase Deficiency causes reduced proliferation of cells under conditions of limited asparagine	10.1016/j.ymgme.2015.08.007	Included: Molecularly confirmed ASNSD clinical report
51	PubMed/MEDLINE; Scopus; Web of Science	Asparagine Synthetase Deficiency with Intracranial Hemorrhage Can Mimic Molybdenum Cofactor Deficiency	10.1055/s-0040-1718917	Included: Molecularly confirmed ASNSD clinical report
52	PubMed/MEDLINE; Web of Science	Asparagine Synthetase Deficiency: Neuropathological Evidence of Disrupted Cortical Development	10.1002/jmd2.70126	Included: Molecularly confirmed ASNSD clinical report
53	PubMed/MEDLINE; Scopus; Web of Science	Asparagine Synthetase Deficiency: New Inborn Errors of Metabolism	10.1007/8904_2014_405	Included: Molecularly confirmed ASNSD clinical report
54	PubMed/MEDLINE; Scopus; Web of Science	Asparagine Synthetase deficiency-report of a novel mutation and review of literature	10.1007/s11011-017-0073-6	Included: Molecularly confirmed ASNSD clinical report
55	PubMed/MEDLINE; Scopus; Web of Science	Asparagine synthetase deficiency detected by whole exome sequencing causes congenital microcephaly, epileptic encephalopathy and psychomotor delay	10.1007/s11011-014-9618-0	Included: Molecularly confirmed ASNSD clinical report
56	PubMed/MEDLINE; Scopus; Web of Science	Asparagine synthetase deficiency: A novel case with an unusual molecular mechanism	10.1016/j.ymgmr.2019.100509	Included: Molecularly confirmed ASNSD clinical report
57	PubMed/MEDLINE; Scopus; Web of Science	Asparagine synthetase deficiency: clinical features and experience with asparagine supplementation	10.1515/jpem-2026-0234	Included: Molecularly confirmed ASNSD clinical report

58	PubMed/MEDLINE; Scopus	Case Report: a novel homozygous ASNS variant in a Chinese female with severe microcephaly, encephalopathy and epilepsy	10.3389/fnins.2025.1570160	Included: Molecularly confirmed ASNSD clinical report
59	PubMed/MEDLINE; Scopus; Web of Science	Case report: A compound heterozygous mutations in ASNS broadens the spectrum of asparagine synthetase deficiency in the prenatal diagnosis	10.3389/fped.2023.1273789	Included: Molecularly confirmed ASNSD clinical report
60	PubMed/MEDLINE; Scopus; Web of Science	Cellular and molecular characterization of two novel asparagine synthetase gene mutations linked to asparagine synthetase deficiency	10.1016/j.jbc.2022.102385	Included: Molecularly confirmed ASNSD clinical report
61	PubMed/MEDLINE; Scopus; Web of Science	Characterization of a novel variant in siblings with Asparagine Synthetase Deficiency	10.1016/j.ymgme.2017.12.433	Included: Molecularly confirmed ASNSD clinical report
62	PubMed/MEDLINE; Scopus; Web of Science	Characterizing asparagine synthetase deficiency variants in lymphoblastoid cell lines	10.1002/jmd2.12356	Included: Molecularly confirmed ASNSD clinical report
63	PubMed/MEDLINE; Scopus	Clinical outcomes of two patients with a novel pathogenic variant in ASNS: response to asparagine supplementation and review of the literature	10.1038/s41439-019-0055-9	Included: Molecularly confirmed ASNSD clinical report
64	PubMed/MEDLINE; Web of Science	Clinical whole exome sequencing from dried blood spot identifies novel genetic defect underlying asparagine synthetase deficiency	10.1002/ccr3.1284	Included: Molecularly confirmed ASNSD clinical report
65	PubMed/MEDLINE; Scopus; Web of Science	Clinical, molecular, and biochemical delineation of asparagine synthetase deficiency in Saudi cohort	10.1038/s41436-020-0919-x	Included: Molecularly confirmed ASNSD clinical report
66	PubMed/MEDLINE; Scopus	Cyst-Peritoneal Shunt for the Treatment of a Progressive Intracerebral Cyst Associated with ASNS Mutation: Case Report and Literature Review	10.1016/j.wneu.2019.02.130	Included: Molecularly confirmed ASNSD clinical report
67	PubMed/MEDLINE; Scopus	Deficiency of asparagine synthetase causes congenital microcephaly and a progressive form of encephalopathy	10.1016/j.neuron.2013.08.013	Included: Molecularly confirmed ASNSD clinical report

68	PubMed/MEDLINE; Scopus; Web of Science	Diaphragmatic Eventration in Sisters with Asparagine Synthetase Deficiency: A Novel Homozygous ASNS Mutation and Expanded Phenotype	10.1007/8904_2016_3	Included: Molecularly confirmed ASNSD clinical report
69	PubMed/MEDLINE; Scopus	Epileptic Phenotype of Two Siblings with Asparagine Synthesis Deficiency Mimics Neonatal Pyridoxine-Dependent Epilepsy	10.1055/s-0036-1586222	Included: Molecularly confirmed ASNSD clinical report
70	PubMed/MEDLINE; Scopus; Web of Science	Epileptic encephalopathy with microcephaly in a patient with asparagine synthetase deficiency: a video-EEG report	10.1684/epd.2019.1100	Included: Molecularly confirmed ASNSD clinical report
71	PubMed/MEDLINE; Scopus	Fetal MRI and ultrasound findings of a confirmed asparagine synthetase deficiency case	10.1002/pd.5772	Included: Molecularly confirmed ASNSD clinical report
72	PubMed/MEDLINE; Scopus; Web of Science	Generation of four induced pluripotent stem cell lines, GZWWTi001-A, GZWTZi001-A, GZWXYi001-A, and GZWXDi001-A, derived from peripheral blood mononuclear cells from a family with asparagine synthetase deficiency	10.1016/j.scr.2019.101583	Included: Molecularly confirmed ASNSD clinical report
73	PubMed/MEDLINE; Scopus	Hyperekplexia, microcephaly and simplified gyral pattern caused by novel ASNS mutations, case report	10.1186/s12883-016-0633-0	Included: Molecularly confirmed ASNSD clinical report
74	PubMed/MEDLINE; Scopus; Web of Science	Ketogenic diet-responsive drug-resistant epilepsy in a case of asparagine synthetase deficiency with a novel compound heterozygous missense variant	10.1016/j.clineuro.2023.107772	Included: Molecularly confirmed ASNSD clinical report
75	PubMed/MEDLINE; Scopus; Web of Science	Molecular diagnosis of asparagine synthetase (ASNS) deficiency in two Indian families and literature review of 29 ASNS deficient cases	10.1016/j.gene.2019.04.024	Included: Molecularly confirmed ASNSD clinical report
76	PubMed/MEDLINE; Scopus; Web of Science	Novel Mutations in the Asparagine Synthetase Gene (ASNS) Associated With Microcephaly	10.3389/fgene.2018.00245	Included: Molecularly confirmed ASNSD clinical report
77	PubMed/MEDLINE; Scopus	Rapid exome sequencing and adjunct RNA studies confirm the pathogenicity of a novel	10.1002/humu.24101	Included: Molecularly confirmed ASNSD clinical report

		homozygous ASNS splicing variant in a critically ill neonate		
78	PubMed/MEDLINE; Scopus; Web of Science	Report of four novel variants in ASNS causing asparagine synthetase deficiency and review of literature	10.1111/cga.12275	Included: Molecularly confirmed ASNSD clinical report
79	PubMed/MEDLINE; Web of Science	Revisiting Exome Data Identified Missed Splice Site Variant of the Asparagine Synthetase (ASNS) Gene	10.1055/s-0042-1757193	Included: Molecularly confirmed ASNSD clinical report
80	PubMed/MEDLINE; Scopus	Severe Microcephaly and Metabolic Epilepsy due to Asparagine Synthetase Deficiency	10.4103/aian.aian_414_23	Included: Molecularly confirmed ASNSD clinical report
81	PubMed/MEDLINE; Scopus; Web of Science	The first report of Japanese patients with asparagine synthetase deficiency	10.1016/j.braindev.2016.09.010	Included: Molecularly confirmed ASNSD clinical report
82	PubMed/MEDLINE; Scopus; Web of Science	The first report of a Korean/Vietnamese child with novel pathogenic variants in Asparagine Synthetase Deficiency (ASNSD) with evolving epilepsy syndromes	10.1016/j.seizure.2023.11.020	Included: Molecularly confirmed ASNSD clinical report
83	PubMed/MEDLINE; Scopus; Web of Science	Worsening of Seizures After Asparagine Supplementation in a Child with Asparagine Synthetase Deficiency	10.1016/j.pediatrneurol.2016.01.024	Included: Molecularly confirmed ASNSD clinical report
84	PubMed/MEDLINE	[Clinical and genetic spectrum of 6 cases with asparagine synthetase deficiency]	10.3760/cma.j.cn112140-20230915-00193	Included: Molecularly confirmed ASNSD clinical report
